# Computed Three-Dimensional Atlas of Subthalamic Nucleus and Its Adjacent Structures for Deep Brain Stimulation in Parkinson's Disease

**DOI:** 10.5402/2012/592678

**Published:** 2012-01-12

**Authors:** Naoki Nakano, Mamoru Taneda, Akira Watanabe, Amami Kato

**Affiliations:** Department of Neurosurgery, Faculty of Medicine, Kinki University, 377-2 Ohno-higashi, Osaka-sayama, Osaka 589-8511, Japan

## Abstract

*Background*. Deep brain stimulation (DBS) of the subthalamic nucleus (STN) is one of the standard surgical treatments for advanced Parkinson's disease. However, it has been difficult to accurately localize the stimulated contact area of the electrode in the subthalamic nucleus and its adjacent structures using a two-dimensional atlas. The goal of this study is to verify the real and detailed localization of stimulated contact of the DBS electrode therapeutically inserted into the STN and its adjacent structures using a novel computed three-dimensional atlas built by a personal computer. *Method*. A three-dimensional atlas of the STN and its adjacent structures (3D-Subthalamus atlas) was elaborated on the basis of sagittal slices from the Schaltenbrand and Wahren stereotactic atlas on a personal computer utilizing a commercial software. The electrode inserted into the STN and its adjacent structures was superimposed on our 3D-Subthalamus atlas based on intraoperative third ventriculography in 11 cases. *Findings*. Accurate localization of the DBS electrode was identified using the 3D-Subthalamus atlas, and its clinical efficacy of the electrode stimulation was investigated in all 11 cases. *Conclusion*. This study demonstrates that the 3D-Subthalamus atlas is a useful tool for understanding the morphology of deep brain structures and for the precise anatomical position findings of the stimulated contact of a DBS electrode. The clinical analysis using the 3D atlas supports the contention that the stimulation of structures adjacent to the STN, particularly the zona incerta or the field of Forel H, is as effective as the stimulation of the STN itself for the treatment of advanced Parkinson's disease.

## 1. Introduction

Deep brain stimulation (DBS) is a proven functional neurosurgical treatment for Parkinson's disease, essential tremor, and generalized dystonia that are intractable with optimal medication [1–4]. The subthalamic nucleus (STN) is considered to be one of the optimal targets for placement of DBS treatment, and STN stimulation has given optimal clinical effect for a wide spectrum of symptoms such as rigidity, tremor, bradykinesia, and gait disturbance [2, 5].

When administering DBS, the position of the stimulated contact of the DBS electrode is one of the important factors for achieving optimal clinical results. However, it is difficult to understand the anatomical relationship between the electrode and the STN based on a two-dimensional atlas. The DBS electrode is inserted via a burr hole made 25–30 mm lateral to the midline of the skull and 20–30 mm anterior to the coronal suture. The angle of the electrode trajectory is about 60 degrees from the horizontal plane of the anterior commissure-posterior commissure line (AC-PC) and 20 degrees from the sagittal plane [6]. The localization of the STN was 8–15 mm lateral to the midline on coronal magnetic resonance imaging (MRI) [7].

 The shape of the STN is convex and ovoid in the Schaltenbrand and Wahren atlas (S-W atlas) [7]. The S-W atlas consists of two-dimensional images expressed in coronal, sagittal, and horizontal sections of the brain relative to the AC-PC line [8]. It is difficult to understand the three-dimensional localization and shape of the STN from two-dimensional views in the atlas, and the shape and location of the STN must be inferred from two-dimensional images. The localization of the DBS electrode in the brain structure cannot be shown in a single sagittal section of the atlas. 

Development of the computers and software has provided a new method for understanding of brain structures and the diagnosis of disease [9–11]. Advanced computer techniques creating 3D views are proving to be useful tools [12, 13]. For example, three-dimensional computed tomography angiography (3D-CTA) is helpful for the diagnosis of lesions in patients with ruptured and nonruptured aneurysms [14].

 In functional surgery, previous reports have demonstrated that 3D computer reconstruction of subcortical brain structures has been helpful for education and for the execution of stereotactic operations [15]. We have devised 3D images of the STN and its adjacent structures and investigated the correlation between the anatomical position of DBS and its clinical effect on patients with Parkinson's disease.

## 2. Methods and Materials

We investigated 11 consecutive patients who received DBS targeting the STN for treatment of advanced Parkinson's disease. Nine women and 2 men were evaluated in the Kinki University Hospital. These patients included the following: one case at 1.5 on the Hoehn and Yahr scale [16] (HY scale) and 2 cases at 2.5, 6 cases at 3, and 2 cases at 4 on the HY scale. The mean age ± standard deviation was 63.5 ± 9.4 years. The mean duration of disease was 10.6 ± 4.6 years (Table 1). All patients received appropriate medical control by neurologists in the Kinki University Hospital prior to the surgical treatment.

Preoperative and postoperative evaluations were performed using the Unified Parkinson's Disease Performance Scale [17] (UPDRS). We examined all patients at one week before surgery during the on-medications period and 6 months after surgery during treatment with DBS and medication. The patients' chief complaints were akinesia (UPDRS II items 9, 10, 11, 12, 13, 14, 15), the “on-off” phenomenon (and the percentage of daily time spent in off states), tremor (UPDRS III items 20 and 21), gait disturbance (UPDRS III items 27, 28, 29, and 30), dyskinesia (and the percentage of daily time spent in dyskinesia), and rigidity (UPDRS III item 22, on the side contralateral to DBS stimulation) (Table 2) [18]. The daily levodopa equivalent dose was calculated on the basis of the following equivalences: 100 mg standard levodopa = 140 mg controlled release levodopa = 10 mg bromocriptine = 1 mg pergolide = 1.5 mg cabergoline = 1 mg pramipexole = 10 mg selegiline [19, 20]. We used the Model 3387 implanted electrode (Medtronic, Inc, USA). This electrode had four contacts 1.5 mm apart. Stimulation was attempted with several parameters in all patients. The parameters consisted of the intensity and amplitude (volts), pulse width (micro seconds), frequency (Hz), and selecting the stimulated contact as a cathode with monopolar stimulation. Postoperative assessment after the DBS showed that the stimulation of one side of bilateral DBS rapidly produced a reduction of tremor, rigidity, and dyskinesia on the contralateral side [21]. We found that the “on-off” phenomenon, on-phase dyskinesia, bradykinesia/akinesia, and gait disturbance were ameliorated after 2 or 3 days using one of 4 electrode contacts. Axial symptoms such as bradykinesia and gait disturbance were improved by bilateral stimulation [22]. All symptoms were evaluated whilst on medication with levodopa. Stimulation-induced adverse effects including dyskinesia, sensory misperception and muscle contraction were also observed with each parameter. We could manage the parameter that reduced motor fluctuation and improved quality of life for all patients.

### 2.1. The Three-Dimensional Atlas

We created a three-dimensional atlas (3D-Subthalamus atlas) based on sagittal slices from the S-W atlas which consisted of the following structures; the nucleus ventrointermedius (VIM), ventralis oralis posterior (VOP) and ventralis oralis anterior (VOA), zona incerta (ZI), the field of Forel H (FH), the red nucleus (RN), substantia nigra (SN), and subthalamic nucleus (STN). 3D-Subthalamus was built from 10 sagittal slices (3.5 mm, 5.5 mm, 6.5 mm, 9.0 mm, 10.5 mm, 12.0 mm, 13.0 mm, 14.0 mm, 16.0 mm, and 17.0 mm lateral to the midline of the original S-W atlas relative to the AC-PC lines). There were two planes of the stained histologic slices and transparent overlay contours on one slice of the S-W atlas [8]. Each structure was traced on the transparent overlay contours of the S-W atlas using 3D software (Rokkakudaiou, Super3, Shusaku, Japan) on a personal computer (Power Book G4, OS 10.3.7, Apple Computer, Inc.). There was a known distance between the slice of each sagittal section and we created a virtual structure by inserting straight lines between the structures of each slice to create a 3D model. All structures were built with reference to the AC-PC line. The 3D-Subthalamus atlas can be viewed from any angle using a computer. The 3D-Subthalamus was created from the S-W atlas so that the AC-PC distance was actually 21 mm in sagittal sections [8]. Therefore, various AC-PC distance (from 20 mm to 26 mm) were prepared to correspond to the individual variations [23, 24]. To allow for these variations in AC-PC distance, all structures were magnified along three axes (sagittal, coronal, and axial planes) using the computer software.

The 3D-Subthalamus atlas is a right-sided model because the S-W atlas was also a right-sided model. Therefore, in the left-sided DBS electrode, the left lateral distance was replaced to right the distance and the position of the left-sided DBS electrodes was investigated on a right-sided model on 3D-Subthalamus atlas.

### 2.2. Surgical Technique

Preoperative MRI provided localization of the subthalamic nucleus, red nucleus, and internal capsule [7, 23, 25, 26]. MRI studies indicate that brain volume and its deep structures have individual variations. For planning the localization of the STN as a target, there are anatomically individual variations of the distance of the STN from the midline [25, 27]. The STN is not so clearly seen on MRI. Bejjani et al. reported that the STN was just lateral to the margin of the RN [25]. The localization of the RN is more easily visualized on MRI than the location of the STN. Therefore, the distance of the RN from midline was defined as the individual lateral distance of the STN in our study.

Under local anesthesia, all patients underwent DBS with selective third ventriculography using the Leksell stereotactic frame (Elekta Instruments, Atlanta, GA). The entry point of the recording electrode was made 20 mm anterior to the coronal suture and approximately 25–30 mm lateral to the midline of the skull. The initial tentative target was 2 mm behind the mid-AC-PC point, 4 mm below this point and 10–12 mm lateral to the midline corresponding to the individual lateral distance on preoperative MRI. To determine the optimal location for final implantation of DBS electrode, electrophysiological recording was used to define the position of the STN using a semi-microelectrode [28]. The semi-microrecording demonstrated specific spontaneous burst potentials on a 4–7 mm line passing through the subthalamic nucleus in most cases [29]. If the duration of recording of the burst potentials was less than 4 mm, we tried to investigate another tentative target within the subthalamic nucleus using the same procedure [29]. Finally, the distal contact of the DBS electrode was positioned at the bottom of the subthalamic nucleus identified by the electrophysiological examination.

 During a week after the stereotactic placement of the DBS electrode, the patient was evaluated for response to the external stimulator settings. Postoperative MRI demonstrated the location of the implanted DBS electrode in the axial, coronal, and sagittal planes. After confirmation of patient satisfaction and optimal localization of the DBS electrode on MRI, bilateral chronic stimulation systems were implanted subcutaneously in the anterior chest wall under general anesthesia in 10 cases. One case with unilateral DBS placement for unilateral Parkinson's symptom was implanted subcutaneously in the left anterior chest wall only.

### 2.3. Investigation of Stimulated Contact of DBS Using the Three-Dimensional Atlas

Postoperative electrode position was determined using an intraoperative ventriculography with a contrast medium and MRI for 10 right-sided and 11 left-sided electrodes. Using 3D software on a personal computer, a 3D model of the DBS electrode was created relative to the AC-PC line. All implanted electrodes were Model 3387 (Medtronic, Inc. USA) which had four contacts with a spacing of 1.5 mm between the contacts. The cathode position of the DBS electrode that provided the optimum clinical effect was investigated on 3D-Subthalamus in all cases.

 There were individual variations in AC-PC distance and the lateral distance of the center of the STN from the midline. The AC-PC distance was measured on ventriculography in each case to correspond to individual variations. Based on the AC-PC distance, the optimal size model was selected from various 3D-Subthalamus atlases, which were prepared with an AC-PC distance of 20–26 mm [30]. The lateral distance from the midline of the STN could not be measured accurately on MRI because of the poor identification of the borderline of the STN [27]. The STN was anatomically identified just lateral to the RN [25]. It was easy to measure the center of the RN because of its spherical shape and its clear identification on T2-weigthed MRI. The lateral distance from the midline of the center of the RN was defined as the distance of the STN and its adjacent structures from the midline. The individual lateral distance and AC-PC distance were applied to the 3D-Subthalamus atlas in each case. For example, in case number 1 the AC-PC distance was 22 mm and RN was located 4.8 mm lateral to the midline on MRI (Table 2). 3D-Subthalamus atlas was adjusted accurately from patient data. Finally, the 3D-Subthalamus atlas with a 22 mm AC-PC distance was conformed using the lateral distance of the RN as measured on MRI for case  1.

## 3. Results

### 3.1. Morphological Evaluation Using the Three-Dimensional Atlas

**Figure 1:**
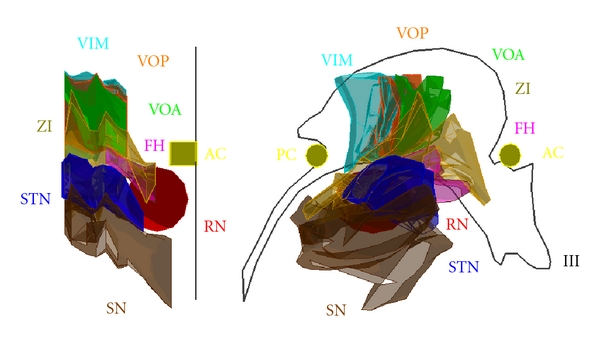
A three-dimensional atlas of the subthalamic nucleus and its adjacent structures (3D-Subthalamus). Left: anterior view. Right: right outside view. VIM: ventrointermedius, VOP: ventralis oralis posterior, VOA: ventralis oralis anterior, ZI: zona incerta, FH: field of Forel H, RN: red nucleus, SN: substantia nigra, STN: subthalamic nucleus, AC: anterior commissure, PC: posterior commissure, and III third ventricle.

**Figure 2:**
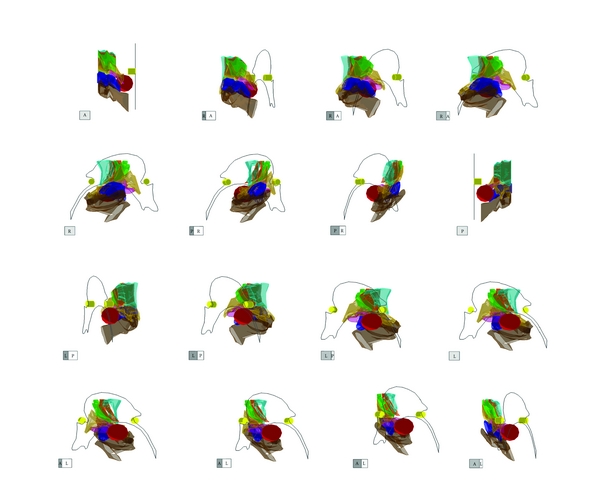
Imaging of the three-dimensional atlas of the subthalamic nucleus and its adjacent structures (3D-Subthalamus). The 3D-Subthalamus is turned 360 degrees along a vertical axis using a computer. Each cube shows the direction of the 3D-Subthalamus atlas. A: anterior, P: posterior, R: right, and L: left.

The shape and relationship of the STN to its adjacent structures could be clearly demonstrated by the three-dimensional atlas (3D-Subthalamus atlas) (Figure 1). Anterior and lateral views show that the STN is located laterally to RN and that the ZI, SN, and FH surround the STN (Figure 1). Though the deep brain structures overlapped each other on 3D-Subthalamus atlas, the 3D-Subthalamus atlas could be freely rotated on the monitor of a personal computer (Figure 2). Observations from various viewpoints allowed us to understand the three-dimensional structures without a blind spot. 

 The STN resembles a bagworm in shape, and its medial part is smaller than its lateral part. The medial surface of the STN is posteroinferior to the RN. The lateral portion of the STN is ampullar in shape and is located posterior to the medial portion (Figure 2).

The ZI is located above the STN and is shaped like a coat covering the STN. The end of the ZI is more posterior than the posterior commissure (Figure 2).

The FH is a small area located anteromedial to the STN. The anteroposterior view shows that the medial surface of the FH is inferior to its lateral surface. The lateral view shows that the FH is covered by the ZI and STN.

### 3.2. Localization of Stimulated Contact

**Figure 3:**
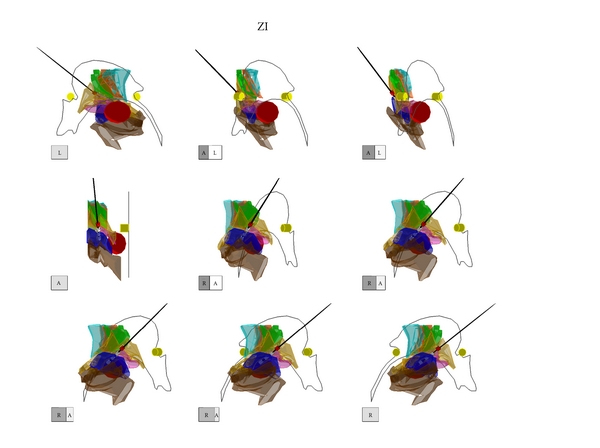
The three-dimensional atlas of the subthalamic nucleus and its adjacent structures (3D-Subthalamus) of an illustrative case (Case  4) of a stimulated contact located in the zona incerta on 3D-Subthalamus. It shows that the contacts of DBS electrode are located at the zona incerta, field of Forel H, and subthalamic nucleus. Best improvement of motor disturbance was obtained when the stimulated contact of DBS electrode was located in the zona incerta. Each cube shows the direction of the 3D-Subthalamus atlas. A: anterior, P: posterior, R: right, and L: left. Red cast: a stimulated contact of the deep brain stimulation electrode. White cast; no stimulated contacts of the deep brain stimulation electrode. Black line: a lead of the deep brain stimulation electrode.

**Figure 4:**
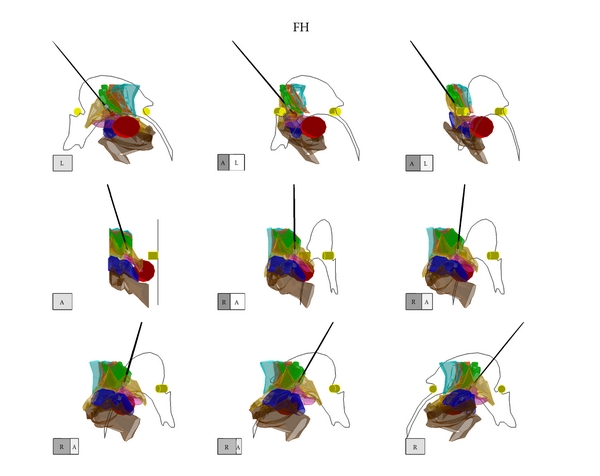
The three-dimensional atlas of the subthalamic nucleus and its adjacent structures (3D-Subthalamus) of an illustrative case (Case  10) of a stimulated contact located in the field of Forel H, on 3D-Subthalamus. It shows that the contacts of DBS electrode are located at the zona incerta, field of Forel H and subthalamic nucleus. Best improvement of motor disturbance was obtained when the stimulated contact of DBS electrode was located in the field of Forel H. Each cube shows the direction of the 3D-Subthalamus atlas. A: anterior, P: posterior, R: right, and L: left. Red cast; a stimulated contact of the deep brain stimulation electrode. White cast: no stimulated contacts of the deep brain stimulation electrode. Black line: a lead of the deep brain stimulation electrode.

**Figure 5:**
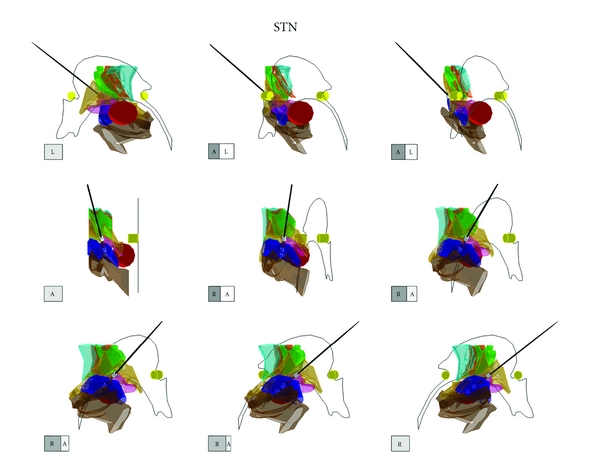
The three-dimensional atlas of the subthalamic nucleus and its adjacent structures (3D-Subthalamus) of an illustrative case (Case  6) of a stimulated contact located in the subthalamic nucleus on the 3D-Subthalamus. It shows that the contacts of DBS electrode are located at the zona incerta, field of Forel H, and subthalamic nucleus. Best improvement of motor disturbance was obtained when the stimulated contact of DBS electrode was located in the subthalamic nucleus. Each cube shows the direction of the 3D-Subthalamus atlas. A: anterior, P: posterior, R: right, and L: left. Red cast: a stimulated contact of the deep brain stimulation electrode. White cast: no stimulated contacts of the deep brain stimulation electrode. Black line: a lead of the deep brain stimulation electrode.

The DBS electrode and its 4 contacts were shown in a three-dimensional figure in the 3D-Subthalamus atlas. The localization of the most effectively stimulated contact (cathode) was investigated using the 3D-Subthalamus atlas to study a correlation between its localization and clinical effect (Figures 3, 4, and 5). The locations of the most effectively stimulated contacts of our 11 patients are shown in Table 2. These locations were divided into three areas that obtained the best improvement of motor disturbance: the ZI in 9 of 21 stimulated contacts (Figure 3), the FH in 6 stimulated contacts (Figure 4), and the STN in another 6 stimulated contacts (Figure 5). 

### 3.3. The Clinical Effect with DBS

Motor function had improved in all patients at 6 months after surgery (Table 2). The UPDRS scores improved in all cases by a mean 54.2% in all cases. Transient sensory disturbance was common but disappeared following the optimal DBS parameter settings. At 6 months after surgery no patients had suffered any severe adverse effects such as cognitive impairment.

The mean lateral distance of the RN from the midline was 5.5 mm ± 0.7 on MRI. The mean AC-PC distance was 22.2 mm ± 1.6 on ventriculography.

### 3.4. The Relationship between Clinical Effect and Location of Stimulated Contact

**Figure 6:**
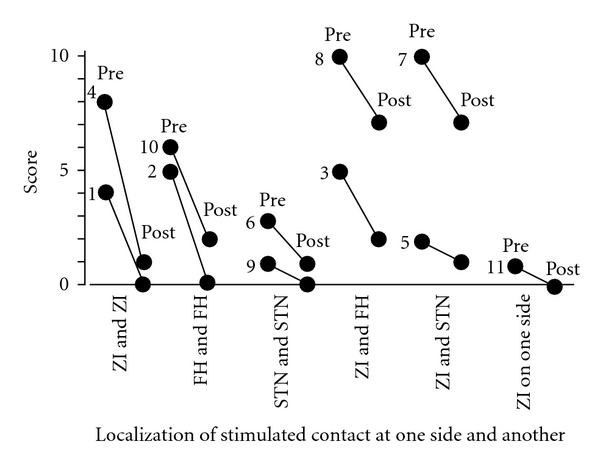
Relationships between the localization of the most effectively stimulated contacts and the UPDRS motor score of items 9, 10, 11, 12, 13, 14, and 15 as akinesia. The best improvement of akinesia was achieved in 10 cases by bilateral deep brain stimulation of each paired combination among the subthalamic nucleus, zona incerta, and field of Forel H and in one case by contralateral stimulation of the zona incerta. Pre: before surgery, post: after surgery, STN: subthalamic nucleus, ZI: zona incerta, FH: field of Forel H. and number: case number.

Akinesia is improved by DBS in all 11 patients. All but one patient were stimulated bilaterally, with the following combinations of stimulated contacts: 2 patients with bilateral ZI stimulation, 2 with bilateral FH stimulation, 2 with bilateral STN stimulation, 2 with combined ZI and FH stimulation, 2 with combined ZI and STN stimulation, and one patient with unilateral ZI stimulation (Figure 6). 

**Figure 7:**
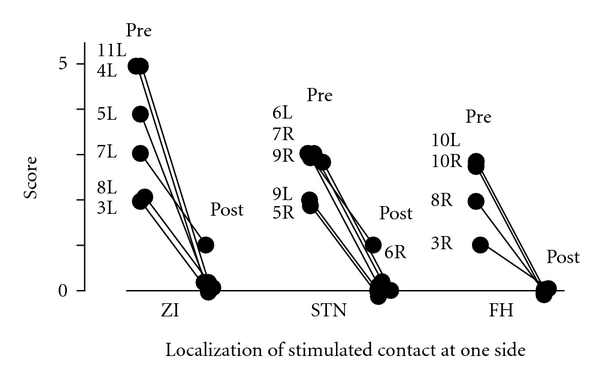
Relationships between the localization of the most effectively stimulated contacts and the UPDRS motor score of items 20 and 21 as tremor. Each case demonstrated the improvement in tremor on one side by deep brain stimulation of the subthalamic nucleus, zona incerta, or field of Forel H on the contralateral side. The preoperative tremor scores in the cases stimulated at the zona incerta are higher than in the cases that were stimulated at the field of Forel H or subthalamic nucleus. Pre: before surgery, post: after surgery, STN: subthalamic nucleus, ZI: zona incerta, FH: field of Forel H, and number and R or L: case number at right (R) or left (L) tremor.

 Of the 16 sides of 11 patients who suffered from tremor preoperatively, 6 had stimulated contacts in the ZI, 4 in the FH, and 6 in the STN. The tremor of all these patients was ameliorated by contralateral DBS, with complete disappearance of the tremor in all patients stimulated at the ZI (Figure 7). The preoperative tremor scores in the cases stimulated at the ZI were higher than in cases who were stimulated at the FH or STN.

There was no statistically significant difference (*P* < 0.05) among the three stimulating points as regards efficacy of DBS for control of tremor.

**Figure 8:**
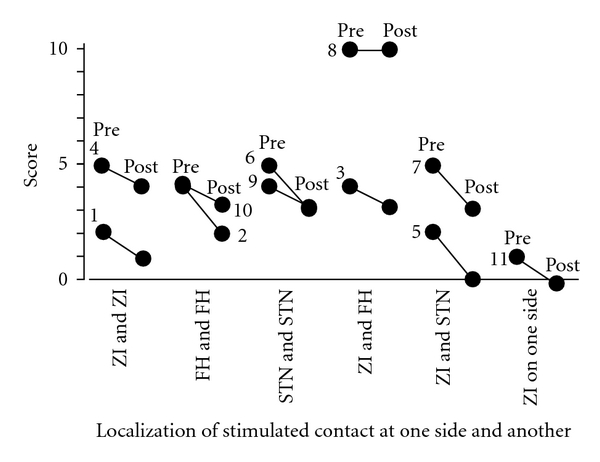
Relationships between the localization of the most effectively stimulated contact and the UPDRS motor score of items 27, 28, 29, and 30 as gait disturbance. The best improvement of gait disturbance was achieved in 9 cases of all 11 cases by bilateral deep brain stimulation of each paired combination among the subthalamic nucleus, zona incerta, and field of Forel H and in one case by unilateral stimulation of the zona incerta. Pre: before surgery, post: after surgery. STN: subthalamic nucleus, ZI: zona incerta, FH: field of Forel H, and number: case number.

 Gait disturbance was ameliorated by the DBS stimulation in all 11 patients with the following combinations of stimulated contacts: 2 with bilateral ZI, 2 with bilateral FH, 2 with bilateral STN, 2 with combined ZI and FH, 2 with combined ZI and STN, and one with unilateral ZI stimulation (Figure 8).

**Figure 9:**
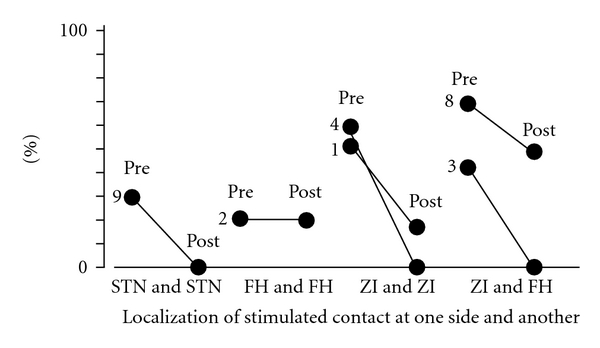
Relationships between the localization of the most effectively stimulated contact and the percentage of daily time spent in off states. All cases except one achieved the best improvement in the on-off phenomenon by bilateral deep brain stimulation of each paired combination of the subthalamic nucleus, zona incerta and field of Forel H. Pre: before surgery, post: after surgery. STN: subthalamic nucleus, ZI: zona incerta, FH: field of Forel H, and number: case number.

 The stimulated contacts of 6 cases with preoperative “on-off” phenomenon were located as follows: one at bilateral STN, one at bilateral FH, 2 at bilateral ZI, and 2 at combination of ZI and FH (Figure 9), and all cases except one bilateral FH stimulation showed reduction of the “on-off” phenomenon.

**Figure 10:**
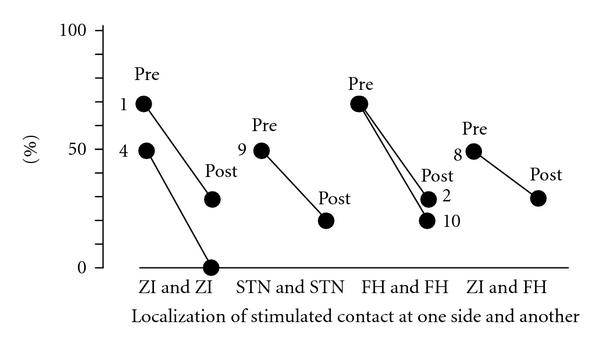
Relationships between the localization of the most effectively stimulated contact and the percentage of daily time spent in dyskinesia in 6 cases that have been affected with dyskinesia before surgery. All cases achieved the best improvement in the on-off phenomenon by bilateral deep brain stimulation of each paired combination of the subthalamic nucleus, zona incerta and field of Forel H. Pre: before surgery, post: after surgery. STN: subthalamic nucleus, ZI: zona incerta, FH: field of Forel H, and number: case number.

 Six sides of dyskinesia were ameliorated by a mean of 21.8%. The stimulated contacts were located as follows: 2 at the ZI, one at the STN, and 3 at the FH (Figure 10). The dyskinesia of 2 of 3 cases stimulated at FH improved postoperatively with unchanged dosage in item the of levodopaequivalents.

## 4. Discussion

### 4.1. The Three-Dimensional Atlas

The aim of this study is to evaluate the relationship between the clinical effect and the localization of the stimulated contact of the DBS electrode for advanced Parkinson's disease. In some studies, the identification and localization of the stimulated contact were based on MRI studies [7, 25, 31–33], and these studies indicated that MRI is a useful tool for preoperative targeting. Recently, some new computer programs such as the Leksell SurgiPlan program (Elekta) have been released for planning the initial targeting. This program compares the patient's MRI coordinates with computerized atlas of deep structures to localize the STN [33]. However, the targeting method of STN still remains somehow a difficult problem, because the STN cannot be convincingly distinguished from its adjacent structures on MRI. The identification of the anterior line and medial borders of the STN can be uncertain on MRI [26]. This is referable to that the shape of the STN is anatomically convex and ovoid on the S-W atlas [8] and to that MRI scans use three planes to visualize the three-dimensional structures; the shape of the STN must be inferred from images in three planes. We found that the three-dimensional atlas is a useful tool for understanding the anatomical relationship of the DBS electrode to the STN and its adjacent structures, regardless of the fact that a two-dimensional atlas is in the sagittal plane and the inserted DBS electrode is not in a consistent plane.

The 3D-Subthalamus is based on the S-W atlas, which is considered to be useful for functional stereotaxic neurosurgery [8]. The S-W atlas consists of the brain sections from 3 different cadavers, because the atlas includes 3 axes: sagittal, coronal, and axial sections [8, 34]. Niemann et al. found that the solid volume of the thalamus differs in the 3 sections of the S-W atlas [34, 35]. 

There are anatomical differences between patients in the AC-PC distance and the lateral distance of the STN from the midline. To correct this, we have designed the 3D-Subthalamus atlas for various AC-PC distances and defined the lateral distance from the midline of the center of the RN as the lateral distance of the STN and its adjacent structures from the midline.

 The localization of the DBS electrode within the STN is generally indicated as corresponding to the sagittal section of the S-W atlas or MRI. However, it is actually impossible to superimpose the stimulated electrode on the corresponding point in the S-W atlas and MRI because the plane including the axis of the electrodes does not always correspond with that of the S-W atlas or MRI. Therefore, it has not been easy to understand the three-dimensional relationship between the target and the electrode. By contrast, the anatomical relationships can be easily understood and clearly observed from any chosen viewpoint using the 3D-Subthalamus atlas.

### 4.2. The Localization of the DBS Electrode on Postoperative MRI

On MRI for postoperative evaluation of the DBS electrode, the tip of the electrode is distorted and enlarged by magnetic artifact [36]. There is a safety limit to the use of MRI for the neurostimulator system (including the DBS electrode and the battery) [37, 38]. MRI-related heating is a major problem for the neurostimulator and for human tissues [26, 37]. In our experience, when performing an MRI sequence to localize the STN, minimizing electrode artifact is incompatible with clearly detecting STN. Open MRI is considered useful as a real-time navigator in neurosurgery [39] but is too expensive for general use.

Therefore, to evaluate the postoperative anatomical localization of the DBS electrode, a reliable method is required by which patient data can be coordinated with an anatomical atlas without the MRI artifact [7]. This method transfers the patient's electrode position onto the corresponding slice from the atlas based on the AC-PC system. Individual variations in each case are generally disregarded in this method. Talairach and Tournoux devised a unique method to correlate the individual variations in the height, length, and width of human brains [40]. This method has been named the “three-dimensional proportional grid system” which adapts to varying dimensions of brains in three planes: the AC-PC line, a vertical line traversing the AC, and a sagittal plane parallel to the interhemispheric plane [40]. Our three-dimensional atlas is similar in that the patient data are registered along three planes based on the AC-PC distance for individual variations. The authors defined the lateral distance from the midline of the STN and its adjacent structures based on the localization of the RN from midline. Yelnik et al. showed that registration of a 3D atlas with MRI allows evaluation of anatomical localization of DBS using the atlas and that correction for individual differences required scaling along the two axes: the mediolateral and anteroposterior axes [7]. The three-dimensional software used in our study produces the scaling model along the three axes on a personal computer. This software is not expensive (about US80 dollar), and no special tools or special technique are required to use it. 

### 4.3. Anatomy of the STN and Its Adjacent Structures

The STN is considered to be one of the most suitable targets for DBS in advanced Parkinson's disease with motor fluctuation that persists despite adequate medical therapy [1, 2, 5, 20, 22, 41]. Interventions involving the STN include subthalamotomy and STN-DBS [42]. STN-DBS has advantages and has fewer complications than subthalamotomy. DBS parameter can be adjusted according to variations in Parkinsonian symptoms. Different positions of the stimulated contact of the DBS electrodes result in various clinical effects [43]. In our study all stimulated contacts of the DBS electrodes within the STN were located within the anterodorsal part of the STN. Neuroanatomical studies have shown that nigral efferent fibers originating from the pars compacta project into the dorsal part of the STN [28, 43, 44]. These fibers finally enter the corpus striatum. Our findings suggest that the most effective point of contact within the STN is located in the dorsal part of the STN. 

 The neighboring structures of the STN are the VIM, VOP, VOA, ZI, FH, RN, and the STN. The ZI and FH are also considered to be valid targets for the stimulation in advanced Parkinson's disease [7, 43].

 The ZI has multiple afferent pathways running into the ventrolateral thalamus which involve the lenticulothalamic system from the mesencephalic reticular formation, the cerebellothalamic tract, and the pallidothalamic tracts [45]. Murata et al. demonstrated that 8 patients with severe tremor were improved by stimulation of the posterior part of the subthalamic white matter (ZI) [45]. This clinical study revealed that the axons could be more easily excited by electrical stimulation than the cell bodies themselves [45]. High-frequency stimulation of the STN induced an increased firing rate in certain thalamic neurons [46]. DBS mainly acts upon axonal elements, and therefore ZI stimulation has similar effectiveness as STN stimulation for Parkinson's disease [47]. These studies suggested that the stimulation of the ZI having the axons causes more excitation of the thalamus than the stimulation of the STN having the cell bodies.

 Stimulation of the STN and globus pallidum internum (GPi) is effective for Parkinson's disease with dyskinesia [48]. The GPi stimulation directly improves dyskinesia, and STN stimulation allows reduction of levodopa dosage: STN stimulation does not directly improve dyskinesia in patients with Parkinson's disease. In our study, two cases showed improved dyskinesia without reduction of levodopa dosage following stimulation of the FH. These results suggest that FH stimulation has a direct influence on dyskinesia. This may be explained by the fact that the FH contains axons of the pallidothalamic tract [43]. Our results emphasize that the FH is the optimal target for Parkinson's disease with dyskinesia.

 While the mechanism of action of DBS remains poorly understood, the different clinical effects induced by stimulation of various targets may provide us with more clear understanding about the mechanisms. Our results from ZI and FH stimulation suggest that a small diameter axon is more excitable by electrical stimulation than the cell bodies [3, 47].

From our results, both the ZI and the FH were shown to be optimal targets for DBS for Parkinson's disease. However, there are anatomical and electrophysiological problems when targeting them. It is difficult to identify the ZI and FH on MRI, and electrophysiological targeting requires skilled technique and refined instruments [49–51]. Some reports have shown that the characteristic electrophysiological activity could not be identified in the FH and ZI [25, 44, 45, 52].

### 4.4. The Adverse Effects of DBS

STN stimulation may induce transient or permanent side effects [3, 53]. Transient side effects can be ameliorated by adjustment stimulation parameters. Several groups have reported permanent side effects including psychiatric problem such as depression or mania [51, 54–59]. These psychiatric problems may be influenced by the limbic circuit following placement of a DBS electrode. Depression may occur with reduction of levodopa after DBS. The anterior ventral STN is anatomically involved in the limbic circuit [52, 60]. Therefore, stimulation of the STN may produce psychiatric side effects.

 Our results show that the stimulation of the ZI or FH is nearly equivalent in effect to the stimulation of the STN for motor fluctuation in Parkinson's disease. Stimulation of the ZI or FH has not triggered psychiatric problems in any case after DBS surgery. Our study indicates that stimulation of the ZI or FH is more effective than stimulation of the STN for Parkinson' s disease and is without severe side effects. Our results are supported by previous studies which found that stimulation of the adjacent structures to the STN improves Parkinsonian symptoms [7, 43]. 

### 4.5. The DBS Electrode

Our study shows that the stimulation of the ZI or FH located above the STN is effectives for advanced Parkinson's disease. The Model 3387 DBS electrode, which is longer than Model 3389, was used in our study. The upper one or two contacts of the Model 3387 can be located above the STN; whenever the lower contact of the Model 3387 DBS electrode is placed in STN, the upper one or two contacts can be located above the STN on 3D-Subthalamus atlas. This means that structures adjacent to the STN can be stimulated with this model as required. Therefore, we recommend the long DBS electrode (model 3387) for advanced Parkinson's disease. No adverse effects were detected in all cases following placement of this model 3387 electrode.

## 5. Conclusion

A three-dimensional atlas of the STN and its adjacent structures (3D-Subthalamus atlas) has been created using the commercial software on a personal computer, which does not require any special techniques. This software is not expensive and is already in current use. 3D-Subthalamus atlas revealed that the localization of DBS electrode could be precisely deduced on the STN and its adjacent structures and that the morphological understanding of the STN and its adjacent structures was promoted by the computed images.

Results demonstrated that the stimulated contacts were located in the STN and/or in its adjacent structures such as the ZI and FH. Stimulation of structures adjacent to the STN was more effective than STN stimulation in cases with tremor and dyskinesia. Specifically, ZI stimulation could improve tremor and FH stimulation could improve dyskinesia without a decrease in levodopa dosage.

On analysis of computed three-dimensional atlas of stimulated electrode and deep brain structures, we emphasize that 3D-Subthalamus atlas is a useful tool to recognize the complex shape of deep brain structures for stereotactic surgery and that the ZI or FH is one of the main targets for DBS in the treatment of patients of Parkinson's disease manifesting severe tremor and/or dyskinesia.

## Figures and Tables

**Table 1:** Summary of the main characteristics of 11 patients with Parkinson's disease.

Case no.	Age (yrs)	Sex	Preoperative Hoehn and Yahr stage (medication on period)	Duration of Parkinson's disease (yrs)
1	42	F	2.5	8
2	66	F	2.5	8
3	66	F	3	12
4	61	F	4	20
5	73	M	3	10
6	61	F	3	10
7	68	F	3	3
8	77	F	3	14
9	54	F	4	14
10	64	F	3	12
11	66	M	1.5	6
Mean	63.5		3.0	10.6
± S.D.	9.4		0.7	4.6

Mean ± SD: mean ± standard deviation.

**Table 2:** The chief complaints, clinical effects, and anatomical locations of the stimulated contacts in 11 patients treated with deep brain stimulation.

Case no.	UPDRS		Localization of stimulated contact		AC-PC (mm)	RN (mm)	Akinesia		Tremor				Gait disturbance		On-Off (%/day)		Dyskinesia (%/day)		Levodopa equivalent (mg/day)	
			Right	Left					Right		Left									
	Pre	Post					Pre	Post	Pre	Post	Pre	Post	Pre	Post	Pre	Post	Pre	Post	Pre	Post
1	19	10	ZI	ZI	22	4.8	4	0	0	0	0	0	2	1	50	20	70	30	565	468
2	26	9	FH	FH	22	6.0	5	0	0	0	0	0	4	2	20	20	70	30	347	280
3	28	16	ZI	FH	21	6.0	5	2	1	0	2	0	4	3	40	0	0	0	533	418
4	31	15	ZI	ZI	21	6.0	8	1	0	0	5	0	5	4	60	0	50	0	628	400
5	19	4	ZI	STN	20	6.0	2	1	2	0	4	0	2	0	0	0	0	0	342	271
6	28	12	STN	STN	23	4.0	3	1	3	1	3	0	5	3	0	0	0	0	396	253
7	44	25	ZI	STN	24	6.0	10	7	3	0	3	1	5	3	0	0	0	0	557	590
8	73	60	ZI	FH	25	5.4	10	7	2	0	2	0	10	10	70	50	50	20	503	503
9	24	7	STN	STN	21	5.6	1	0	3	0	2	0	4	3	30	0	50	20	570	500
10	44	16	FH	FH	21	6.0	6	2	3	0	3	0	4	3	0	0	70	20	285	285
11	13	4	ZI	No placement	24	5.0	1	0	0	0	5	0	1	0	0	0	0	0	440	307
Mean	31.7	16.5			22.2	5.5	5	1.9	1.5	0.1	2.6	0.1	4.2	2.9	24.5	8.2	32.7	10.9	470	388.6
± S.D.	16.7	15.6			1.6	0.7	3.3	2.6	1.4	0.3	1.7	0.3	2.4	2.7	27.0	16.0	32.3	13.0	113.5	116.1

Akinesia: UPDRS II items 9, 10, 11, 12, 13, 14, 15, on-off and percentage of daily time spent in off states, tremor: UPDRS III items 20 and 21, gait disturbance: UPDRS III items 27, 28, 29, and 30, dyskinesia: the percentage of daily time spent in dyskinesia, rigidity: UPDRS III item 22 on the side contralateral to DBS stimulation.

AC-PC: distance of anterior commissure-posterior commissure, RN: lateral distance from the midline of red nucleus, ZI: zona incerta, STN: subthalamic nucleus, FH: field of forel H, and mean ± S.D.: mean ± standard deviation, pre: before surgery, post: after surgery.

## References

[1] Benabid AL, Benazzouz A, Hoffmann D, Limousin P, Krack P, Pollak P (1998). Long-term electrical inhibition of deep brain targets in movement disorders. *Movement Disorders*.

[2] Kleiner-Fisman G, Fisman DN, Sime E, Saint-Cyr JA, Lozano AM, Lang AE (2003). Long-term follow up of bilateral deep brain stimulation of the subthalamic nucleus in patients with advanced Parkinson disease. *Journal of Neurosurgery*.

[3] Lozano AM (2001). Deep brain stimulation for Parkinson’s disease. *Parkinsonism and Related Disorders*.

[4] Olanow CW, Brin MF, Obeso JA (2000). The role of deep brain stimulation as a surgical treatment for Parkinson’s disease. *Neurology*.

[5] Krack P, Batir A, Van Blercom N (2003). Five-year follow-up of bilateral stimulation of the subthalamic nucleus in advanced Parkinson’s disease. *The New England Journal of Medicine*.

[6] Perozzo P, Rizzone M, Bergamasco B (2001). Deep brain stimulation of the subthalamic nucleus in Parkinson’s disease: comparison of pre- and postoperative neuropsychological evaluation. *Journal of the Neurological Sciences*.

[7] Yelnik J, Damier P, Demeret S (2003). Localization of stimulating electrodes in patients with Parkinson disease by using a three-dimensional atlas-magnetic resonance imaging coregistration method. *Journal of Neurosurgery*.

[8] Schaltenbrand G, Wahren W (1977). *Atlas for Stereotaxy of the Human Brain*.

[9] Alesch F, Koos WT (1995). Computer-Assisted multidimensional atlas for functional stereotaxy. *Acta Neurochirurgica*.

[10] Kikinis R, Gleason PL, Moriarty TM (1996). Computer-assisted interactive three-dimensional planning for neurosurgical procedures. *Neurosurgery*.

[11] St-Jean P, Sadikot AF, Collins L (1998). Automated atlas integration and interactive three-dimensional visualization tools for planning and guidance in functional neurosurgery. *IEEE Transactions on Medical Imaging*.

[12] Kazarnovskaya MI, Borodkin SM, Shabalov VA, Krivosheina VY, Golanov AV (1991). 3-D computer model of subcortical structures of human brain. *Computers in Biology and Medicine*.

[13] Kelemen A, Székely G, Gerig G (1999). Elastic model-based segmentation of 3-D neuroradiological data sets. *IEEE Transactions on Medical Imaging*.

[14] González-Darder JM, Pesudo-MartÍnez JV, Feliu-Tatay RA (2001). Microsurgical management of cerebral aneurysms based in CT angiography with three-dimensional reconstruction (3D-CTA) and without preoperative cerebral angiography. *Acta Neurochirurgica*.

[15] Yoshida M (1993). Three-dimensional electrophysiological atlas created by computer mapping of clinical responses elicited on stimulation of human subcortical structures. *Stereotactic and Functional Neurosurgery*.

[16] Hoehn MM, Yahr MD (1967). Parkinsonism: onset, progression and mortality. *Neurology*.

[17] Fahn S, Elton RL, Members of the UPDRS Development Committee, Fahn S, Marsden CD, Goldstein M, Calne DB (1987). The unified Parkinson’s disease rating scale. *Recent Development in Parkinson’s Disease*.

[18] Robertson LT, Horak FB, Anderson VC, Burchiel KJ, Hammerstad JP (2001). Assessments of axial motor control during deep brain in Parkinsonian patients. *Neurosurgery*.

[19] Lozano AM, Lang AE, Galvez-Jimenez N (1995). Effect of GPi pallidotomy on motor function in Parkinson’s disease. *The Lancet*.

[20] Vingerhoets FJG, Villemure JG, Temperli P, Pollo C, Pralong E, Ghika J (2002). Subthalamic DBS replaces levodopa in Parkinson’s disease: two-year follow-up. *Neurology*.

[21] Germano IM, Gracies JM, Weisz DJ, Tse W, Koller WC, Olanow CW (2004). Unilateral stimulation of the subthalamic nucleus in Parkinson disease: a double-blind 12-month evaluation study. *Journal of Neurosurgery*.

[22] Kumar R, Lozano AM, Sime E, Halket E, Lang AE (1999). Comparative effects of unilateral and bilateral subthalamic nucleus deep brain stimulation. *Neurology*.

[23] DiPierro CG, Francel PC, Jackson TR, Kamiryo T, Laws ER (1999). Optimizing accuracy in magnetic resonance imaging-guided stereotaxis: a technique with validation based on the anterior commissure-posterior commissure line. *Journal of Neurosurgery*.

[24] Peppe A, Pierantozzi M, Bassi A (2004). Stimulation of the subthalamic nucleus compared with the globus pallidus internus in patients with Parkinson disease. *Journal of Neurosurgery*.

[25] Bejjani BP, Dormont D, Pidoux B (2000). Bilateral subthalamic stimulation for Parkinson’s disease by using three-dimensional stereotactic magnetic resonance imaging and electrophysiological guidance. *Journal of Neurosurgery*.

[26] Richter EO, Hoque T, Halliday W, Lozano AM, Saint-Cyr JA (2004). Determining the position and size of the subthalamic nucleus based on magnetic resonance imaging results in patients with advanced Parkinson disease. *Journal of Neurosurgery*.

[27] Starr PA, Christine CW, Theodosopoulos PV (2002). Implantation of deep brain stimulators into the subthalamic nucleus: technical approach and magnetic resonance imaging-verified lead locations. *Journal of Neurosurgery*.

[28] Rodriguez-Oroz MC, Rodriguez M, Guridi J (2001). The subthalamic nucleus in Parkinson’s disease: somatotopic organization and physiological characteristics. *Brain*.

[29] Sterio D, Zonenshayn M, Mogilner AY (2002). Neurophysiological refinement of subthalamic nucleus targeting. *Neurosurgery*.

[30] Benabid AL, Koudsie A, Benazzouz A, Le Bas JF, Pollak P (2002). Imaging of subthalamic nucleus and ventralis intermedius of the thalamus. *Movement Disorders*.

[31] Cuny E, Guehl D, Burbaud P, Gross C, Dousset V, Rougier A (2002). Lack of agreement between direct magnetic resonance imaging and statistical determination of a subthalamic target: the role of electrophysiological guidance. *Journal of Neurosurgery*.

[32] Starr PA, Vitek JL, DeLong M, Bakay RAE (1999). Magnetic resonance imaging-based stereotactic localization of the globus pallidus and subthalamic nucleus. *Neurosurgery*.

[33] Yoon MS, Munz M (2000). Placement of deep brain stimulators into the subthalamic nucleus. *Stereotactic and Functional Neurosurgery*.

[34] Niemann K, Naujokat C, Pohl G, Wollner C, Keyserlingk VD (1994). Verification of the Schaltenbrand and Wahren stereotactic atlas. *Acta Neurochirurgica*.

[35] Niemann K, Van Nieuwenhofen I (1999). One atlas—three anatomies: relationships of the Schaltenbrand and Wahren microscopic data. *Acta Neurochirurgica*.

[36] Rezai AR, Finelli D, Nyenhuis JA (2002). Neurostimulation systems for deep brain stimulation: in vitro evaluation of magnetic resonance imaging-related heating at 1.5 Tesla. *Journal of Magnetic Resonance Imaging*.

[37] Rezai AR, Phillips M, Baker KB (2004). Neurostimulation system used for deep brain stimulation (DBS): MR safety issues and implications of failing to follow safety recommendations. *Investigative Radiology*.

[38] Rezai AR, Sharan A, Nyenhuis JA (2003). MR safety in patients with implanted deep brain stimulation systems (DBS). *Acta Neurochirurgica, Supplementum*.

[39] De Salles AAF, Frighetto L, Behnke E (2004). Functional neurosurgery in the MRI environment. *Minimally Invasive Neurosurgery*.

[40] Talairach J, Tournoux P (1988). *Co-Planar Stereotactic Atlas of the Human Brain*.

[41] Pahwa R, Wilkinson SB, Overman J, Lyons KE (2003). Bilateral subthalamic stimulation in patients with Parkinson disease: long-term follow up. *Journal of Neurosurgery*.

[42] Su PC, Tseng HM, Liu HM, Yen RF, Liou HH (2002). Subthalamotomy for advanced Parkinson disease. *Journal of Neurosurgery*.

[43] Voges J, Volkmann J, Allert N (2002). Bilateral high-frequency stimulation in the subthalamic nucleus for the treatment of Parkinson disease: correlation of therapeutic effect with anatomical electrode position. *Journal of Neurosurgery*.

[44] Theodosopoulos PV, Marks WJ, Christine C, Starr PA (2003). Locations of movement-related cells in the human subthalamic nucleus in Parkinson’s disease. *Movement Disorders*.

[45] Murata JI, Kitagawa M, Uesugi H (2003). Electrical stimulation of the posterior subthalamic area for the treatment of intractable proximal tremor. *Journal of Neurosurgery*.

[46] Benazzouz A, Gao DM, Ni ZG, Piallat B, Bouali-Benazzouz R, Benabid AL (2000). Effect of high-frequency stimulation of the subthalamic nucleus on the neuronal activities of the substantia nigra pars reticulata and ventrolateral nucleus of the thalamus in the rat. *Neuroscience*.

[47] McIntyre CC, Savasta M, Kerkerian-Le Goff L, Vitek JL (2004). Uncovering the mechanism(s) of action of deep brain stimulation: activation, inhibition, or both. *Clinical Neurophysiology*.

[48] Nutt JG, Rufener SL, Carter JH (2001). Interactions between deep brain stimulation and levodopa in Parkinson’s disease. *Neurology*.

[49] Guridi J, Rodriguez-Oroz MC, Lozano AM (2000). Targeting the basal ganglia for deep brain stimulation in Parkinson’s disease. *Neurology*.

[50] Henderson JM, Dunnett SB (1998). Targeting the subthalamic nucleus in the treatment of Parkinson’s disease. *Brain Research Bulletin*.

[51] Romito LM, Raja M, Daniele A (2002). Transient mania with hypersexuality after surgery for high-frequency stimulation of the subthalamic nucleus in Parkinson’s disease. *Movement Disorders*.

[52] Starr PA, Theodosopoulos PV, Turner R (2003). Surgery of the subthalamic nucleus: use of movement-related neuronal activity for surgical navigation. *Neurosurgery*.

[53] Monaca C, Ozsancak C, Defebvre L (2004). Transient insomnia induced by high-frequency deep brain stimulation in Parkinson disease. *Neurology*.

[54] Berney A, Vingerhoets F, Perrin A (2002). Effect on mood of subthalamic DBS for Parkinson’s disease: a consecutive series of 24 patients. *Neurology*.

[55] Doshi PK, Chhaya N, Bhatt MH (2002). Depression leading to attempted suicide after bilateral subthalamic nucleus stimulation for Parkinson’s disease. *Movement Disorders*.

[56] Funkiewiez A, Ardouin C, Caputo E (2004). Long term of bilateral subthalamic nucleus stimulation on cognitive function, mood, and behaviour in Parkinson's disease. *Journal of Neurology, Neurosurgery and Psychiatry*.

[57] Hershey T, Revilla FJ, Wernle A, Gibson PS, Dowling JL, Perlmutter JS (2004). Stimulation of STN impairs aspects of cognitive control in PD. *Neurology*.

[58] Kulisevsky J, Berthier ML, Gironell A, Pascual-Sedano B, Molet J, Parés P (2002). Mania following deep brain stimulation for Parkinson’s disease. *Neurology*.

[59] Saint-Cyr JA, Trépanier LL, Kumar R, Lozano AM, Lang AE (2000). Neuropsychological consequences of chronic bilateral stimulation of the subthalamic nucleus in Parkinson’s disease. *Brain*.

[60] Okun MS, Raju DV, Walter BL (2004). Pseudobulbar crying induced by stimulation in the region of the subthalamic nucleus. *Journal of Neurology, Neurosurgery and Psychiatry*.

